# The ATP synthase inhibition induces an AMPK-dependent glycolytic switch of mesenchymal stem cells that enhances their immunotherapeutic potential

**DOI:** 10.7150/thno.51631

**Published:** 2021-01-01

**Authors:** Rafael Contreras-Lopez, Roberto Elizondo-Vega, Noymar Luque-Campos, María José Torres, Carolina Pradenas, Gautier Tejedor, María José Paredes-Martínez, Ana María Vega-Letter, Mauricio Campos-Mora, Yandi Rigual-Gonzalez, Karina Oyarce, Magdiel Salgado, Christian Jorgensen, Maroun Khoury, María de los Ángeles Garcia-Robles, Claudia Altamirano, Farida Djouad, Patricia Luz-Crawford

**Affiliations:** 1 Centro de Investigación e Innovación Biomédica, Facultad de Medicina, Universidad de los Andes, Santiago, Chile.; 2 IRMB, Université de Montpellier, INSERM, Montpellier, France.; 3 Laboratorio de Biología Celular, Departamento de Biología Celular, Facultad de Ciencias Biológicas, Universidad de Concepción, Concepción, Chile.; 4 Escuela de Ingeniería Bioquímica, Pontificia Universidad Católica de Valparaíso, Valparaíso, Chile.; 5 Cells for Cells, Regenero, Las Condes, Santiago, Chile.; 6 Laboratory of Nano-Regenerative Medicine, Facultad de Medicina, Universidad de los Andes, Santiago, Chile.; 7 Facultad de Ciencias de la Salud, Universidad San Sebastián, Concepción, Chile.

**Keywords:** MSC, ATP synthase inhibition, glycolytic metabolism, AMPK activity, immunotherapy

## Abstract

**Objectives:** Mesenchymal Stem/Stromal Cells (MSC) are promising therapeutic tools for inflammatory diseases due to their potent immunoregulatory capacities. Their suppressive activity mainly depends on inflammatory cues that have been recently associated with changes in MSC bioenergetic status towards a glycolytic metabolism. However, the molecular mechanisms behind this metabolic reprogramming and its impact on MSC therapeutic properties have not been investigated.

**Methods:** Human and murine-derived MSC were metabolically reprogramed using pro-inflammatory cytokines, an inhibitor of ATP synthase (oligomycin), or 2-deoxy-D-glucose (2DG). The immunosuppressive activity of these cells was tested *in vitro* using co-culture experiments with pro-inflammatory T cells and *in vivo* with the Delayed-Type Hypersensitivity (DTH) and the Graph versus Host Disease (GVHD) murine models.

**Results:** We found that the oligomycin-mediated pro-glycolytic switch of MSC significantly enhanced their immunosuppressive properties *in vitro*. Conversely, glycolysis inhibition using 2DG significantly reduced MSC immunoregulatory effects. Moreover, *in vivo*, MSC glycolytic reprogramming significantly increased their therapeutic benefit in the DTH and GVHD mouse models. Finally, we demonstrated that the MSC glycolytic switch effect partly depends on the activation of the AMPK signaling pathway.

**Conclusion:** Altogether, our findings show that AMPK-dependent glycolytic reprogramming of MSC using an ATP synthase inhibitor contributes to their immunosuppressive and therapeutic functions, and suggest that pro-glycolytic drugs might be used to improve MSC-based therapy.

## Introduction

Mesenchymal Stem/Stromal Cells (MSC) are potent immunoregulatory cells that are among the best cell candidates for the treatment of inflammatory and autoimmune diseases 1. However, some discrepancies between the results obtained in pre-clinical and clinical studies indicate that MSC immunoregulatory properties need to be thoroughly studied to optimize MSC-based therapies 2.

Indeed, the expansion of human MSC *in vitro*, which is needed for their use in the clinic, promotes their metabolic reprogramming towards oxidative phosphorylation (OXPHOS) that reduces their therapeutic efficacy 3-5. Conversely, MSC culture in an inflammatory microenvironment favors their glycolytic reprogramming and enhances their immunoregulatory potential 4, 6. This differential MSC metabolic reprogramming upon culture in different conditions and exposure to stimuli has direct consequences on their properties, including proliferation, aging, differentiation, and immunosuppression. Indeed, glycolysis inhibition significantly represses MSC immunoregulatory properties by inhibiting the activity of indoleamine 2,3-dioxygenase (IDO), a well-known mediator of the immunosuppressive functions of human MSC 4, 7. However, the role of MSC metabolism in their therapeutic properties and the molecular mechanisms underlying the pivotal role of glycolysis in MSC immunoregulatory functions require to be better understood.

AMP-activated protein kinase (AMPK) is a cellular energy sensor that triggers the molecular pathways needed to supply ATP in response to low ATP levels 8. It is also involved in MSC survival and proliferation in hypoxic culture conditions 9. AMPK activation is associated with increased membrane translocation of glucose transporters 1 and 4 (GLUT1) and (GLUT4) 10. Downstream of AMPK, other signaling pathways, such as mTOR, also regulate glucose homeostasis 11, 12. The induction of glucose uptake and glycolysis downstream mTOR depends on the transcription factor hypoxia-inducible factor 1α (HIF1α) 13. In hypoxic conditions, HIF1α increases monocarboxylate transporter-4 (MCT4) expression in cancer cells, consistent with the high rate of glycolysis and the need to export high quantity of lactic acid in the extracellular environment 14. Similarly, glycolysis and high expression levels of MCT4 are critical for the macrophage response 15. Our group has recently demonstrated that HIF1α has a central role in the regulation of MSC glycolytic reprogramming and immunoregulatory properties; however, it is not known whether other metabolic mediators, such as AMPK, play a role in MSC functionality. Therefore, in the present study, we evaluated the impact of MSC metabolic changes on their immunoregulatory and therapeutic properties, and investigated the underlying mechanisms by focusing on AMPK role. To this aim, we analyzed the energetic profile of MSC in inflammatory conditions that activates their immunoregulatory properties. Additionally, we induced metabolic switches by incubating MSC with anti- and pro-glycolytic drugs. Then, we investigated *in vitro* the functional consequences of mouse and human MSC metabolic reprogramming using T-cell proliferation tests. Finally, we tested *in vivo* the therapeutic effect of metabolically reprogrammed MSC in two mouse models of delayed-type hypersensitivity (DTH) and humanized graft versus host disease (GVHD).

## Material and Methods

### MSC isolation and culture

Murine MSC were isolated from C57BL6 mice and characterized as previously described 16. Human MSC derived from umbilical cord (UC-MSC), menstrual blood (MenSC), and bone marrow (BM-MSC) were isolated, expanded and fully characterized, as previously described 17-19. All the experiments with human MSC were performed at early passages (P4-P8). All MSC were cultured in Dulbecco's modified eagle medium (DMEM) high glucose (Corning, USA) supplemented with 10% fetal bovine serum (FBS), 1% penicillin/streptomycin and 1% glutamine (Gibco, Thermo Fisher, USA), at 37 ºC and 5% CO_2_ in normoxia. When indicated, both murine MSC and UC-MSC were stimulated for 24 h with 20 ng/mL of interferon-γ (IFNγ) and 10 ng/mL of tumor necrosis factor-α (TNFα) (R&D Systems, USA), or with 1 mM 2-deoxy-D-glucose (2DG) for murine MSC or 5 mM 2DG for human MSC (Sigma-Aldrich, Merck, Germany), or with 1 μg/mL of oligomycin (Calbiochem, Merck, Germany). AMPK activity was inhibited with 10 μM of compound C (Calbiochem, Merck, Germany). All the procedures presented in this work were carried according to the US National Institutes of Health Guide for the Care and Use of Laboratory Animals (NIH Publication No. 85-23, revised 1996), and were approved by the Institutional Animal Care and Use Committee of the Universidad de los Andes, Santiago, Chile (Number 201630) and in agreement with the Ethical Committee for animal experimentation of the Languedoc- Roussillon (Approval CEEA-LR-2016050918509993).

### Immunosuppression assay

CD4^+^ T cells were freshly isolated from spleen of C57BL/6 mice by negative selection with the Dynabeads Untouched Mouse CD4 Cells Kit (Invitrogen, Thermo Fisher, USA) according to the manufacturer's instructions. Once purified, cells were labeled with CellTrace Violet (CTV) (Life-Technology, Thermo Fisher, USA) and activated with CD3/CD28 beads (Invitrogen, Thermo Fisher, USA). Lymphocytes were cultured in mixed lymphocyte reaction (MLR) medium, containing 10% FBS, 1% penicillin/streptomycin, 1% sodium pyruvate, 1% non-essential amino acids, 1% glutamine and 25 µM β-mercaptoethanol (Gibco, Thermo Fisher, USA), in Iscove's Modified Dulbecco's Medium (IMDM) (Gibco, Thermo Fisher, USA). For differentiation towards the Th1 subtype, purified CD4^+^ T cells were stimulated with 10 ng/mL of IL12 (R&D Systems, USA) and 2.5 μg/mL of anti-IL4 antibodies (BD Pharmingen, BD Biosciences, USA). Differentiation towards the Th17 phenotype was induced with 50 ng/mL of IL6 (R&D Systems, USA), 2.5 ng/mL of TGFβ1 (R&D Systems, USA), and 2.5 μg/mL of anti-IFNγ (BD Pharmingen, USA) and 2.5 μg/mL of anti-IL4 antibodies (BD Pharmingen, USA).

To assess the immunosuppressive properties of murine MSC, CD4^+^ T cells were cultured alone or in the presence of MSC at a cell ratio of 1 MSC per 10 lymphocytes in MLR medium. After 72 h, CD4^+^ T cell proliferation and differentiation were quantified by flow cytometry.

For human MSC immunosuppressive assays, peripheral blood mononuclear cells (PBMC) were isolated from fresh blood samples donated by healthy donors, with Ficoll-Paque PLUS density gradient (GE Healthcare, USA). Isolated PBMC were stained with CTV and stimulated with 5 µg/mL of phytohemagglutinin-L (PHA; Sigma-Aldrich, Merck, Germany) and cultured in MLR medium for 4 days, with or without BM-MSC, MenSC, or UC-MSC at a ratio of 1 to 20 or 1 to 50 for UC-MSCs, as indicated in the figure legends.

### Flow cytometry

Lymphocyte proliferation and differentiation were quantified by flow cytometry. T cells were stimulated with phorbol myristate acetate (PMA) (50 ng/mL; Merck, Germany) and ionomycin (1 mg/mL; Merck, Germany), in the presence of brefeldin A (10 mg/mL; Sigma, Merck, Germany) for 4 h. Then, cells were incubated with an antibody against CD25 (BD Pharmingen) and stained with the LIVE/DEAD Fixable near-IR stain (Invitrogen, Thermo Fisher, USA) to analyze only live cells. Then, cells were fixed at 4 °C with the FOXP3 Cytofix/Cytoperm buffer (eBioscience, USA) and stained with intracellular fluorochrome-conjugated antibodies against IFN-γ (BD Pharmingen), IL-17 (BD Pharmingen) and FOXP3 (eBioscience) diluted in Perm/Wash buffer (eBioscience, USA) according to the manufacturer's specifications.

### Metabolic measurements

Oxygen consumption rate (OCR) and extracellular acidification rate (ECAR) were quantified using the Seahorse XF96 analyzer (Seahorse Biosciences, North Billerica, MA, USA), associated with oxidative phosphorylation and secretion of lactic acid as a metabolic product of glycolysis, respectively. Pre-stimulated murine MSC (20,000 cells/ well) or UC-MSC (12,500 cells/well) were plated on 96-well plates and analyzed according to the manufacturer's recommended protocol. Three independent readings were taken after each sequential injection. The instrument background was measured in separate control wells using the same conditions but without biologic material.

The basal glycolytic rate was calculated after glucose injection (after subtracting the ECAR rate inhibited by 2DG). The maximum glycolytic rate was measured after oligomycin injection and the glycolytic capacity as the difference of oligomycin-induced ECAR and 2DG-induced ECAR. OCR was measured in XF medium (non-buffered DMEM medium, containing 25 mM glucose, 2 mM L-glutamine, and 1mM sodium pyruvate) in basal conditions and in response to 1 μM oligomycin, 1 μM of carbonylcyanide-4-(trifluoromethoxy)-phenylhydrazone (FCCP) and 1 μM of antimycin A and rotenone (all chemicals from Sigma Aldrich). Basal OCR was calculated as the difference between the baseline measurements and the antimycin A/rotenone-induced OCR. The maximum OCR was the difference between the FCCP-induced OCR and antimycin A/rotenone-induced OCR.

### DTH mouse model

To assess the effect of MSC metabolic switch on the generation of different subpopulations of CD4^+^ T lymphocytes* in vivo*, a DTH inflammation model was used. DTH experiments were performed in accordance with the Languedoc-Roussillon Ethical Committee for animal experimentation (Approval CEEA-LR-2016050918509993). Complete Freund's adjuvant and albumin from chicken egg white (ovalbumin; Sigma-Aldrich, Merck, Germany) were injected in the lower back of BALB/c mice. Five days later a boost injection of ovalbumin was done directly in the hindlimb paws, concomitantly with 2x10^5^ MSC or PBS (control group). Paw swelling was measured 24 h after the boost and then mice were euthanized. Blood and draining lymph nodes were analyzed by flow cytometry to identify/quantify the CD4^+^ T lymphocyte populations.

### Humanized xenogeneic GVHD mouse model

NOD-scid IL2rcnull (NSG) mice from The Jackson Laboratory (Bar Harbor, ME, USA) were kept in the specific pathogen-free animal facility of Universidad de los Andes with water and food ad libitum, according to the international guidelines for animal care and the protocols approved by the Institutional Animal Care and Use Committee (Folio CEC Number 201630, Universidad de los Andes, Santiago, Chile). When 10-12-week-old, mice were irradiated (2Gy) at the Chilean Commission for Nuclear Energy Facilities. After 24 h, 12×10^6^ human PBMC (obtained from buffy coats of healthy donors as described above) were injected in the tail (day 0). Two doses of 1×10^6^ MSC or PBS (control group) were injected intraperitoneally at day 0 and day 5. GVHD onset occurs typically 7 days post-PBMC injection and the body weight of the mice was monitored daily. Mice were euthanized when the total body weight loss was >20% of their baseline weight.

### Immunofluorescence

Murine MSC were grown on poly-L-lysine-coated (Sigma-Aldrich, St. Louis, MO, USA) glass coverslips in 24-well plates. Then, they were incubated with 20 ng/mL IFNγ and 10 ng/mL TNFα (R&D Systems, USA), or 1 mM 2DG (Sigma-Aldrich, Merck, Germany), or 1 μg/mL oligomycin (Calbiochem, Merck, Germany) for 24 h. Cell were fixed with 4% paraformaldehyde in PBS for 30 min, washed with Tris-HCl buffer (pH 7.8), and incubated in the same buffer with 1% bovine serum albumin and 0.2% Triton X-100 at room temperature for 10 min. Cells were then incubated with rabbit anti-GLUT2 (1:200, Alomone Labs), rabbit anti-MCT1 (1:100 Merck Millipore, MA, USA) and rabbit anti-MCT4 (1:100, Merck Millipore, MA, USA) antibodies. After incubation with Cy2-labeled secondary antibodies (1:200, Jackson ImmunoResearch Laboratories), cells were counterstained with the DNA stain TOPRO-3 (1:1000, Invitrogen), and analyzed using a confocal laser microscope (Carl Zeiss, LSM700).

### Immunoblotting

Total protein extracts were obtained from MSC cultures. Cells were lysed in RIPA buffer supplemented with protease inhibitor cocktail (ROCHE), and sonicated three times on ice at 300W. Proteins were resolved by SDS-PAGE (50 μg/lane) in 12% (w/v) polyacrylamide gels, transferred to PVDF membranes (0.45 μm pore, Amersham Pharmacia Biotech, Piscataway, NJ, USA), and probed at 4 °C with different antibodies (Table S1) for 16 h. After extensive washes, membranes were incubated with peroxidase-labeled anti-rabbit IgG (1:7000; Jackson ImmunoResearch, West Grove, PA, USA) or rabbit anti-chicken IgY (1:1000; Jackson ImmunoResearch Laboratories, Inc., PA, USA) for 24 h, followed by the enhanced chemiluminescence (ECL) western blot analysis system (Amersham Biosciences, Pittsburgh, PA, USA). Images are representative of at least four immunoblotting analyses performed with samples from at least four separate experiments. β-actin expression level was used as a loading control for densitometric analyses.

### Reverse Transcription-Polymerase Chain Reaction (RT-PCR) and real-time quantitative PCR (qPCR)

RT-PCR and real-time qPCR were performed as previously described 20. Briefly total RNA from cell cultures was isolated using TRIzol™ (Invitrogen, USA) and then incubated with DNase I (Invitrogen) before RT. For RT, 2 µg RNA/sample was incubated in a 20 µL reaction volume containing 10× buffer for M-MuLV reverse transcriptase (New England BioLab, USA), 20U RNAse inhibitor (New England BioLab, USA), 1mM dNTPs, 0.5 µg/µL random primers (Promega, USA), and 200 U M-MuLV reverse transcriptase (New England BioLab, USA) at 37 °C for 5 min, at 42 °C for 60 min and at 70 °C for 10 min. For real-time qPCR, reactions were prepared with Hot FIREPol® DNA polymerase (Solis Biodyne, Estonia) to a final volume of 20 µL containing 2 µL cDNA diluted 1:1 and 500 nM primer (Table S2), and carried out in an Mx3000P QPCR System (Agilent Technologies, USA). Thermal cycling conditions were: 10 min denaturation at 95 °C, followed by 40 cycles of denaturation at 95 °C for 30 s, annealing at 55 °C for 20 s, and extension at 72 °C for 20 s. The relative changes in gene expression were calculated with the relative quantification method (2^-ΔΔCt^) and normalized according to the expression in basal conditions.

### Quantification of immunosuppressive molecules produced by MSC

The expression level of the immunosuppressive molecules TGFβ1, COX2 and IDO was quantified by real time qPCR analysis. To this aim, UC-MSC or murine MSC were plated in 6-well plated and stimulated with TNFα and IFNγ with or without 2DG or oligomycin for 24 h. Total RNA extraction, cDNA synthesis, and qPCR were performed as previously described. Moreover, IDO levels were measured by quantification of L-kynurenine in UC-MSC, using an enzymatic assay as previously described 19. PGE_2_ level in murine MSC and human UC-MSC was quantified with an ELISA Kit (Thermo Fisher, USA) according to the manufacturer's instructions. PD-L1 (BD biosystem, USA) and GLUT1 (Metafora, FR) expression levels were measured in murine MSC by FACS.

### Metabolites quantification

Glucose, lactate, pyruvate and ammonia concentrations were measured using an Analyzer Y15 (BioSystems S.A., Spain) and the D-Glucose/D-Fructose (#12800), Pyruvate (#12826), L-Lactic Acid (#12802) and Ammonia (#12809) kits (BioSystems S.A., Spain). Glutamine concentration was measured with an YSI 2700 Biochemistry Analyzer (Yellow Springs Inc., USA).

### Metabolic mathematical modeling

Metabolic Flux Analysis (MFA) of the intracellular flux of metabolites in different conditions was carried out using the MetaFluxNet 1.8 software. The stoichiometric metabolic model was constructed to explain the MSC metabolic changes after each treatment. The model has 24 reactions and 20 metabolites. It includes reactions of glycolysis, tricarboxylic acids and oxidative phosphorylation. The experimental quantification data of four different metabolites (glucose, lactate, ammonia and glutamine) were used to determine the specific consumption or production rate and feed the model.

### Statistical analysis

Results were expressed as the mean ± SD. All *in vitro* experiments were performed at least four times using four different biological replicates. For the *in vivo* studies (DTH and GVHD models), 8 to 10 animals were used for each experimental or control group, and experiments were repeated at least three independent times. The *p* values were generated by non-parametric analysis using the Kruskal-Wallis test for multiple comparisons and the Mann-Whitney U test to compare two groups; *p* < 0.05 (*), *p* < 0.01 (**) or *p* < 0.001 (***) were considered statistically significant. All the analyses were performed using the GraphPad Prism TM 6 software (GraphPad Software, San Diego, California, USA).

## Results

### Inflammation triggers MSC immunosuppressive properties and induces their glycolytic reprogramming

**Figure 1:**
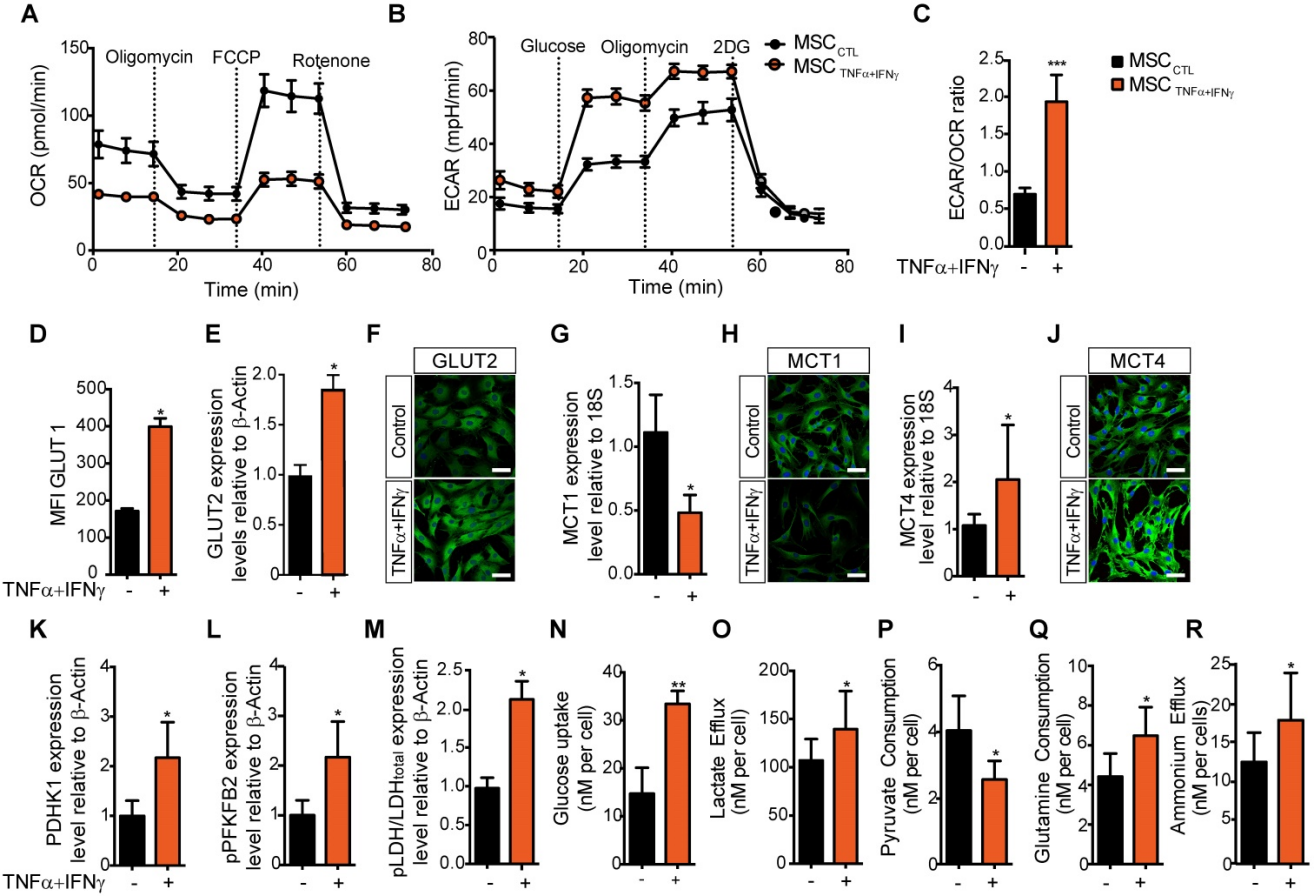
** MSC immunosuppressive activity is associated with a switch towards a glycolysis-dependent metabolism. (A-C)** The metabolic profile of MSC incubated (orange) or not (black) with pro-inflammatory cytokines for 24 h was evaluated by measuring the oxygen consumption rates (OCR)** (A)**, the extracellular acidification rate (ECAR) of the medium **(B)**, and the ECAR/OCR ratio **(C)** using the Agilent Seahorse XF technology. Data are the mean ± SD of at least 4 independent experiments; *** p <0.001 (unpaired Mann-Whitney test). **(D-O)** The expression, uptake, consumption or efflux of enzymes and metabolites associated with glycolysis or OXPHOS was determined in MSC incubated or not with TNFα and IFNγ for 24 h. The expression levels of the specific glucose transporters GLUT1 **(D)** and GLUT2 were quantified by FACS and western blotting **(E),** or immunofluorescence **(F)** analysis. The expression levels of the monocarboxylate transporters MCT1 **(G, H)** and MCT4 **(I, J)** were quantified by qPCR or immunofluorescence. The expression levels of enzymes associated with glycolysis, **(K)** PDHK1 and **(L)** phosphorylated (p) PFKFB2 and **(M)** pLDH, were quantified by western blotting. **(N)** 2-deoxy-D-glucose (2DG) radioactive uptake in cultured MSC was assessed using 10 mM deoxyglucose **(O)** Lactate efflux, **(P)** pyruvate consumption rate, **(Q)** glutamine consumption rate and **(R)** ammonium efflux were quantified from the supernatants of murine MSC activated or not with TNFα and IFNγ using an YSI analyzer. Data are the mean ± SD of at least 4 independent experiments; *: *p* < 0.05, **: *p* < 0.01 (unpaired Mann-Whitney test).

We and others have shown that MSC activation with pro-inflammatory cytokines 21, 22, particularly TNFα and IFNγ, to mimic the pro-inflammatory environment, triggers the release of mediators of MSC immunoregulatory properties and their immunosuppressive potential 23. Here, to determine whether inflammatory cytokines affect MSC metabolism, we compared MSC metabolic activity in basal culture conditions and after incubation with TNFα and IFNγ for 24 h. MSC activation with pro-inflammatory cytokines significantly reduced their basal and maximal OCR and their spare respiratory capacity (SRC), and increased the ECAR in MSC supernatant (Figure 1A-B and Figure S1). The significant increase of the ECAR/OCR ratio of activated MSC compared with naive MSC (Figure 1C) indicated a switch towards glycolysis. To test whether the expression of glucose transporters in MSC was upregulated by exposure to inflammatory stimuli, we measured the expression level of active GLUT1 (by flow cytometry using the Glut1-H2RBD-EGFP fusion protein that detects the active GLUT1 transporter present on MSC membranes) and total GLUT2 (by western blotting and immunofluorescence). Our results showed a significant increase of GLUT1 translocation to the cell membrane (Figure 1D) and of GLUT2 expression level upon stimulation with TNFα and IFNγ (Figure 1E-F and Figure S2A).

### The TNFα and IFNγ-induced glycolytic switch is associated with increased lactate export and expression of glycolytic enzymes

To test whether the glycolytic switch induced by pro-inflammatory cytokines was associated with the activity of the lactate-proton symporters, we evaluated the expression levels of monocarboxylate transporter (MCT) 1 and MCT4 in MSC by qPCR and immunofluorescence. MCT1 expression was significantly decreased (Figure 1G-H), whereas MCT4 mRNA and protein expression levels were significantly increased in MSC incubated with pro-inflammatory cytokines compared with naïve MSC (Figure 1I-J). This suggested an increase in the lactate export to the extracellular space, associated with the higher Km of MCT4 24. Moreover, western blotting showed that the expression of some enzymes associated with glycolysis (Figure S2B-D) was significantly increased: pyruvate dehydrogenase kinase isoform 1 (PDHK1) (Figure 1K) and the active phosphorylated form of phosphorylated-phosphofructokinase 2 (pPFKFB2) (Figure 1L) and phosphorylated Lactate dehydrogenase A (pLDH-A) (Figure 1M).

We then investigated whether this TNFα and IFNγ-induced glycolytic activity in MSC was associated with changes in the consumption of some metabolites or modification in the release of some products and metabolites. First, we observed that after 24 h incubation with TNFα and IFNγ, glucose uptake by MSC was increased (from 17.6 to 33.4 nM/cell) (Figure 1N), as well as lactate efflux (from 107 to 139 nM/cell) (Figure 1O). Conversely, pyruvate consumption was decreased (from 4.0 to 2.4 nM/cell) (Figure 1P), whereas glutamine consumption and ammonium efflux increased (from 4.4 to 6.8 nM/cell and from 12.3 to 17.4 nM/cell, respectively) (Figure 1Q-R).

### MSC metabolic flexibility is confirmed using pharmaceutical inhibitors

**Figure 2:**
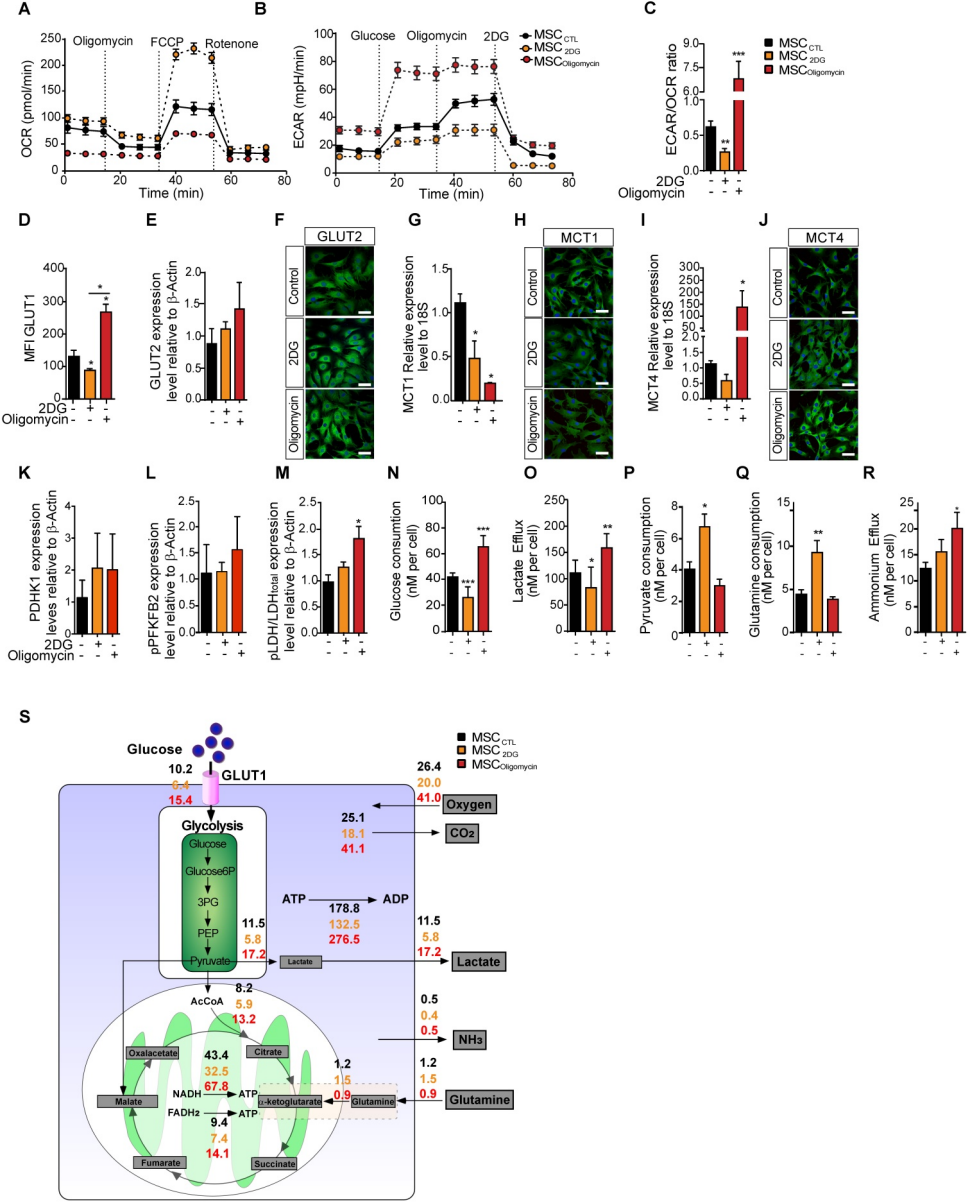
** In MSC, oligomycin and 2DG induce opposite metabolic profiles that replicate or prevent the metabolic switch observed upon classical cytokine stimulation.** The metabolic profile of control MSC (black) or incubated with 2DG (yellow) or oligomycin (red) for 24h was evaluated by measuring the OCR** (A)**, ECAR **(B)** and ECAR/OCR ratio **(C)** using the Agilent Seahorse XF technology. Data are the mean ± SD of at least 4 independent experiments; **: *p* < 0.01, *** *p* < 0.001, **** *p* < 0.0001 (unpaired Mann-Whitney test). Components of the molecular pathways associated with glycolysis or OXPHOS were analyzed in MSC incubated or not with 2DG or oligomycin for 24 h. The expression levels of the specific glucose transporters GLUT1 **(D)** and GLUT2 were quantified by FACS and western blotting** (E)** and immunofluorescence **(F)**. The expression levels of MCT1 **(G, H)** and MCT4 **(I, J)** were quantified by qPCR and immunofluorescence. The expression levels of the glycolysis-associated enzymes PDHK1 **(K)**, phosphorylated (p) PFKFB2 **(L)** and p-LDH **(M)** were quantified by western blotting. Glucose consumption** (N)**, lactate efflux** (O),** pyruvate consumption** (P)**, glutamine consumption** (Q)** and ammonium efflux **(R)** were quantified from the supernatants of murine MSC incubated or not with 2DG or oligomycin using an YSI analyzer. Data are the mean ± SD of at least 4 independent experiments; *: *p* < 0.05, **: *p* < 0.01, *** *p* < 0.001 (unpaired Kruskal-Wallis test). **(S)** Metabolic flux analysis by mathematical modelling of glycolysis, Krebs cycle and oxidative phosphorylation reactions that represent the cellular metabolism state.

To understand the effect of MSC metabolic status on their immunosuppressive functions, we modified MSC metabolic activity by inducing a metabolic switch by incubation with oligomycin (to inhibit OXPHOS) or with 2DG (to inhibit glycolysis). Different concentrations of these drugs were tested to assess their effect on MSC viability, without showing apoptosis induction (data not shown). MSC metabolism monitoring showed that after incubation with oligomycin (MSC_oligomycin_), OCR was decreased and ECAR was increased compared with untreated control MSC (MSC_CTL_). Incubation with 2DG (MSC_2DG_) induced the opposite switch towards enhanced oxygen consumption and reduced ECAR (Figure 2A-B). The ECAR/OCR ratio confirmed that oligomycin induced an overall switch towards a glycolysis-dependent metabolism, and 2DG towards an OXPHOS-dependent metabolism (Figure 2C). In agreement, GLUT1 membrane translocation was significantly increased in MSC_oligomycin_, and significantly decreased in MSC_2DG_ compared with MSC_CTL_ (Figure 2D). GLUT2 protein level was slightly, but not significantly increased in MSC_oligomycin_ (Figure 2E-F). MCT1 expression level was significantly decreased in both MSC_oligomycin_ and MSC_2DG_ compared with MSC_CTL_ (Figure 2G-H). Conversely, MCT4 expression was significantly increased in MSC_oligomycin_ and was reduced in MSC_2DG_ (Figure 2I-J). These results indicated that GLUT1 and MCT4 might play a key role in MSC metabolic switch induced by oligomycin and 2DG. Similarly to the results obtained in MSC stimulated with TNFα and IFNγ, the expression of glycolytic enzymes was increased in MSC_oligomycin_, including a slight increase of PDHK1 (Figure 2K), pPFKFB2 (Figure 2L) and a significant increase of pLDH (Figure 2M) (representative western blot of all enzymes in Figure S2). Additionally, in MSC_oligomycin_ (red bar) glucose uptake (Figure 2N), lactate production (Figure 2O) and ammonium efflux (Figure 2R) were increased and pyruvate consumption (Figure 2P) was decreased. Glutamine consumption was not affected (Figure 2Q). In MSC_2DG_ (dark red bar), glucose uptake and lactate efflux were decreased, whereas pyruvate and glutamine consumption were increased (Figure 2N-R). Using MFA, a mathematical model of cell metabolism (Figure 2S) that included glycolysis, tricarboxylic acids and oxidative phosphorylation reactions, we established that in association with the amount of glucose consumed by MSC, 56% and 45% of the produced pyruvate was converted to lactate in MSC_oligomycin_ and MSC_2DG_, respectively. We also established a link between glutamine consumption and glucose consumption that reached 6% and 23% in MSC_oligomycin_ and MSC_2DG_, respectively. Thus, the increase in glucose consumption and lactate production rates, and the decrease in glutamine consumption, which is associated with a lower requirement of metabolic intermediates for the Krebs cycle, showed that oligomycin promotes a glycolytic metabolic state in MSC. Conversely, 2DG induces an oxidative metabolism.

### Metabolic reprogramming dictates MSC immunoregulatory potential *in vitro* and *in vivo*

**Figure 3:**
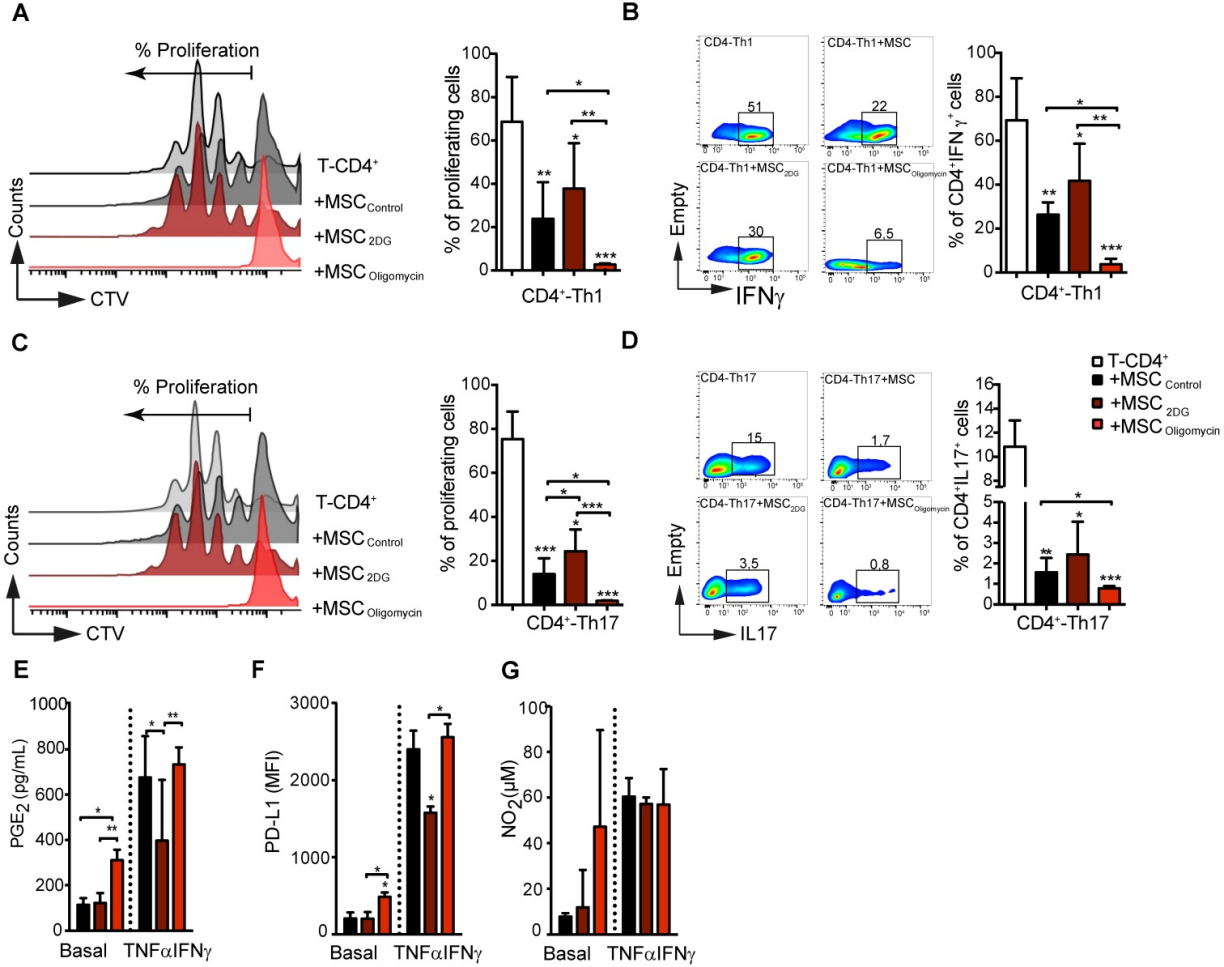
** The immunosuppressive abilities of MSC depend on their metabolic status.** Glycolysis drives MSC suppressive effect on pro-inflammatory Th1 and Th17 cells *in vitro*. Naïve CD4^+^ T cells from Bl6 mice were labeled with CTV and induced to differentiate into Th1 **(A-B)** and Th17 **(C-D)** cells and cultured alone (white bar) or in the presence of control MSC (untreated; black bar) or pre-incubated with oligomycin (red bar) to inhibit OXPHOS, or 2DG (yellow) to inhibit glycolysis. T-cell proliferation and the pro-inflammatory phenotype (IFNγ and IL17 production for Th1 and Th17, respectively) were evaluated by FACS. Results are the mean ± SD of 4 independent experiments with 3 different mice each time; *: *p* < 0.05, **: *p* < 0.01, *** *p* < 0.001 (unpaired Kruskal-Wallis test). Unless otherwise indicated, comparisons were with CD4-Th1 or CD4-Th17 cultured alone. **(E-G)** MSC glycolytic switch increases the production of classical immunosuppressive factors *in vitro*. MSC were incubated or not with oligomycin or 2DG in the presence or absence of TNFα and IFNγ for 24 h, and then the production of NO_2_ **(G)** and PGE_2_ **(E)** was evaluated in the cell supernatants. PD-L1 expression in MSC **(F)** was evaluated by FACS using the geometric mean fluorescence Intensity (MFI) quantification. Results represent the mean ± SD of 4 independent experiments; *:* p* < 0.05, **:* p* < 0.01, *** *p* < 0.001 (unpaired Kruskal-Wallis test). Unless otherwise indicated, comparisons were with MSC in basal conditions.

Then, we assessed the immunoregulatory potential of MSC_oligomycin_ and MSC_2DG_* in vitro* by co-culturing them with freshly isolated mouse CD4^+^ T cells after they were differentiated into Th1 and Th17 cells. T-cell proliferation and phenotype analysis by FACS after 3 days of co-culture, showed that oligomycin greatly enhanced MSC immunosuppressive activity towards Th1 (Figure 3A-B) and Th17 (Figure 3C-D) compared with MSC_CTL_. Conversely, 2DG significantly reduced MSC immunosuppressive capacities (Figure 3A) towards Th1 (Figure 3A-B) and Th17 (Figure 3C-D) cells. Importantly, co-culture with MSC_oligomycin_ and MSC_2DG_ did not affect the generation of Treg cells from naïve CD4^+^ T cells neither induce T-cells apoptosis (data not shown). MSC immunosuppressive activity depends on the production of different molecules, including PD-L1, PGE_2_ and NO_2_, that is stimulated by incubation with TNFα and IFNγ for 24 h 25. Production of PGE_2_ (Figure 3E) and PD-L1 (Figure 3F), but not of NO_2_ (Figure 3G) by MSC_oligomycin_ was already increased in basal conditions compared with MSC_CTL_, and was further enhanced by incubation with TNFα and IFNγ. Conversely, in basal conditions, production of PGE_2_ (Figure 3E), PD-L1 (Figure 3F) and NO_2_ (Figure 3G) was comparable in MSC_2DG_ and MSC_CTL_, and the TNFα/IFNγ-mediated stimulation of PGE_2_ (Figure 3E) and PD-L1 (Figure 3F) production was significantly lower by MSC_2DG_ than MSC_oligomycin_. These results revealed that the glycolytic switch of MSC induced by pro-inflammatory cytokines governs the production of immunosuppressive mediators.

**Figure 4:**
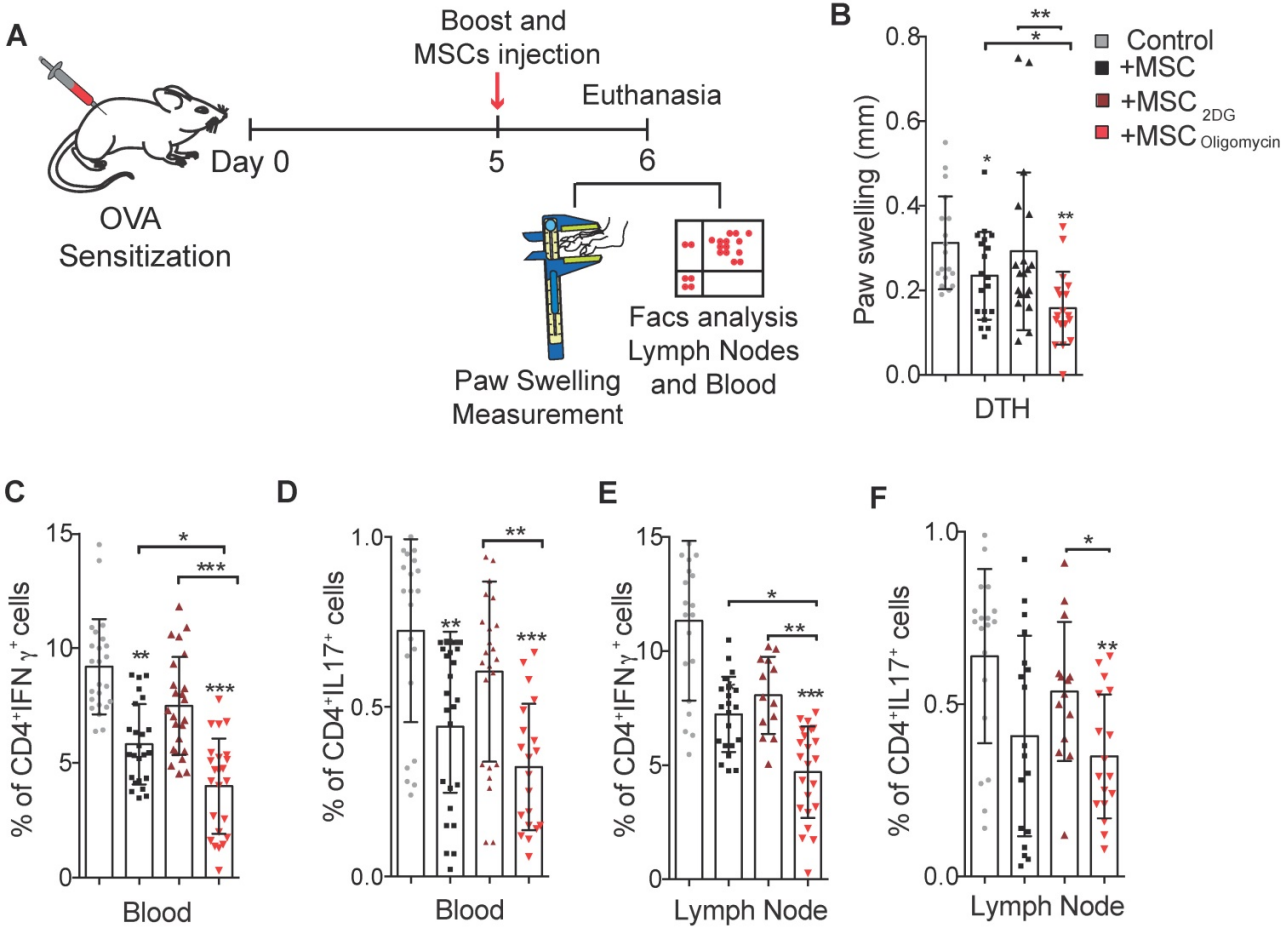
** Glycolytic MSC show enhanced therapeutic potential in a murine arthritic DTH model.** Scheme of the DTH murine model. The boost injection of ovalbumin was performed at the same time as the treatment with MSC or PBS (control) **(A)**. Paw swelling was determined in the hindlimb 24h after the boost and MSC injections **(B)**. The generation of pro-inflammatory T-cells was assessed in peripheral blood **(C, D)** and in draining lymph nodes **(E, F)** of untreated DTH mice (gray) and DTH mice treated with control MSC (black) or MSC pre-incubated with oligomycin (red) or 2DG (orange). Results represent the mean ± SD of 3 independent experiments with at least 10 animals per experimental group; *: *p* < 0.05, **: *p* < 0.01, ***: *p* < 0.001 (unpaired Kruskal Wallis test). Unless otherwise indicated, comparisons were with untreated DTH mice.

To further assess the therapeutic efficacy of MSC_2DG_ and MSC_oligomycin_ we used a mouse model of DTH to evaluate T cell-mediated immune responses 26 (Figure 4A). In mice treated with MSC_oligomycin_ paw swelling was reduced compared with animals that received MSC_2DG_, MSC_CTL_, or no MSC (Figure 4B). This indicated that MSC metabolic switch towards glycolysis enhances their therapeutic and anti-inflammatory functions. This clinical effect was correlated with the significantly lower number of Th1 and Th17 cells detected in peripheral blood (Figure 4C-D) and popliteal lymph nodes (Figure 4E-F) (Representative dot plot in Figure S3) of DTH mice that received MSC_oligomycin_ compared with mice treated with MSC_2DG_ or MSC_CTL_. This effect was not associated with changes in the Treg number in the peripheral blood and popliteal lymph nodes (data not shown). Altogether, these data suggest that the pharmacological modification of MSC metabolism toward glycolysis significantly improves their immunoregulatory abilities *in vitro* and *in vivo*.

### Upon oligomycin incubation, human MSC also switch to glycolysis and show enhanced immunosuppressive capacities

To test the role of the metabolic modulation on the immunosuppressive functions of human MSC, we first incubated human UC-MSC with pro-inflammatory cytokines and then measured their metabolic activity. As observed in murine MSC, UC-MSC activation by TNFα and IFNγ significantly decreased OCR (Figure S4A), and induced glycolysis (Figure S4B). The ECAR/OCR ratio (Figure S4C) and lactate production (Figure S4D) also were significantly increased in UC-MSC activated with TNFα and IFNγ. Moreover, after incubation with 2DG and oligomycin, UC-MSC were metabolically reprogramed toward the OXPHOS and glycolytic metabolism, respectively, as observed with murine MSC. Indeed, OCR was significantly reduced (Figure S4E) and ECAR increased (Figure S4F) in UC-MSC_oligomycin_, compared with control cells (UC-MSC_CTL_). We observed the opposite effect in UC-MSC incubated with 2DG. Consequently, the ECAR/OCR ratio was significantly increased in UC-MSC_oligomycin_ and decreased in UC-MSC_2DG_, compared with UC-MSC_CTL_ (Figure S4G). Lactate production was significantly increased in UC-MSC_oligomycin_, but not in UC-MSC_2DG_ compared with UC-MSC_CTL_ (Figure S4H). Moreover, the glycolytic switch induced by oligomycin was very stable, up to 72 h after its removal from the medium (Figure S4I-K).

**Figure 5:**
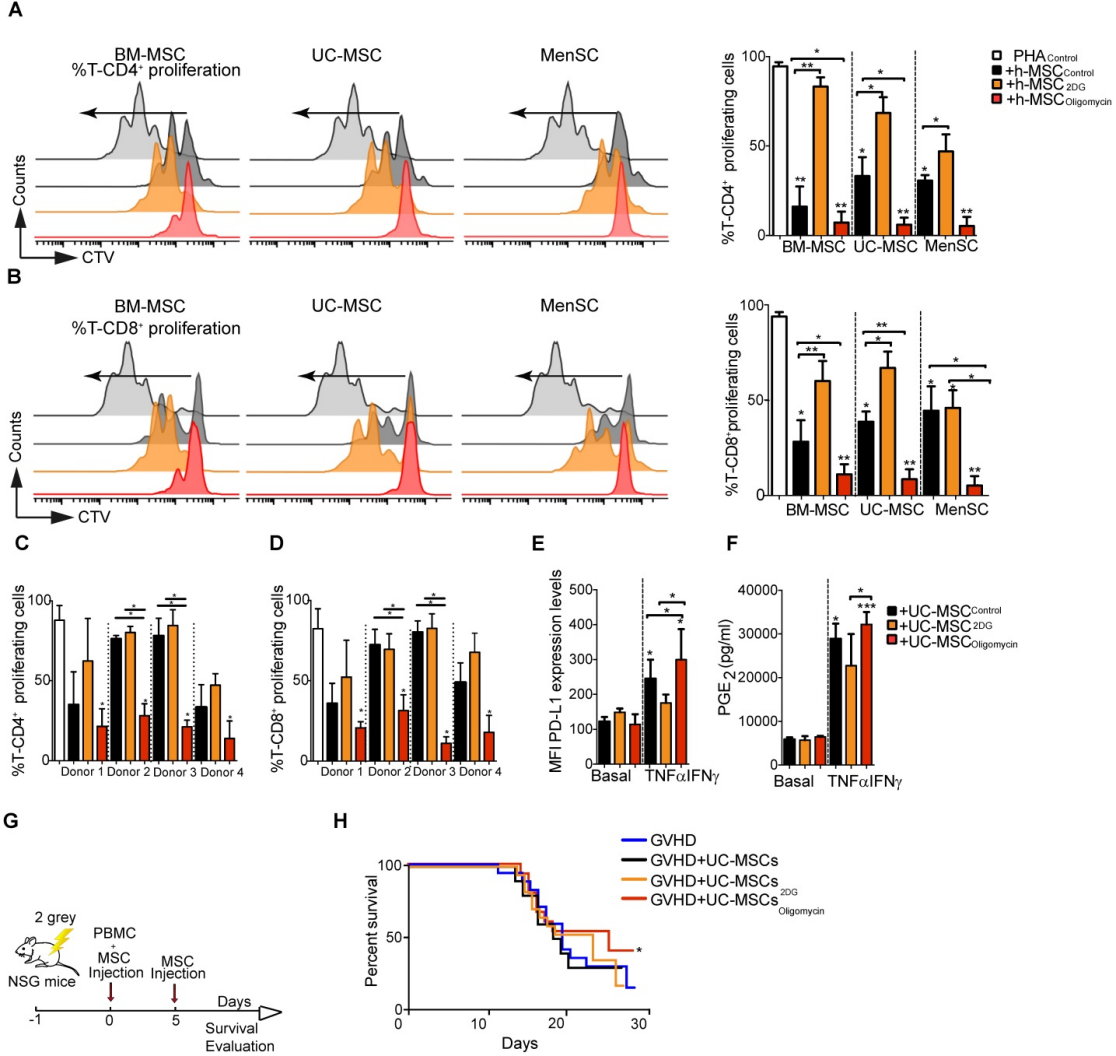
** Oligomycin synchronizes and enhances the immunosuppressive potential of human MSC.** Percentage of proliferating CD4^+^ **(A)** and CD8^+^ T cells **(B)** in PBMC cultured alone or with human MSC derived from bone marrow (BM-MSC), umbilical cord (UC-MSC) and menstrual tissue (MenSC) (different donors) and pre-incubated or not (Control) with 2DG or oligomycin. Cells were co-cultured at a ratio of 1:10 (MSC:PBMC). All MSC types switched to glycolysis displayed enhanced immunosuppressive function**.** Results represent the mean ± SD of 3 independent experiments with 3 different PBMC and 3 MSC donors. The immunosuppressive potential of UC-MSC from different donors, pre-incubated with 2DG or oligomycin, was assessed with PHA-activated human PBMC at a 1:50 ratio (UC-MSC:PBMC) for 4 days. Proliferation of CD4^+^** (C)** and CD8^+^ **(D)** T cells was quantified by FACS. Glycolytic UC-MSC increased the expression of classical immunosuppressive factors *in vitro*, in a pro-inflammatory environment. UC-MSC were pre-incubated or not (black bars) with 1 μg/mL oligomycin (red) or 5 μM of 2DG (orange) in the presence or absence of TNFα and IFNγ for 24 h, and then, PD-L1 expression **(E)** and PGE_2_ production **(F)** were evaluated by FACS and ELISA, respectively. Results represent the mean ± SD of 3 independent experiments with 4 different UC-MSC donors; *: *p* < 0.05, **: *p* < 0.01, ***: *p* < 0.001 (unpaired Kruskal-Wallis test). Unless otherwise indicated, comparisons were with UC-MSC in basal conditions. The glycolytic status of UC-MSC significantly influenced the therapeutic efficacy of UC-MSC in a GVHD model. **(G)** Representative scheme of the GVHD murine model. **(H)** UC-MSC pre-incubated or not with oligomycin were co-injected with PBMC followed by a second infusion of PBMC 5 days later. The mouse weight was monitored daily, and the Kaplan-Meier survival analysis shows the percentage of mice that did not lose more than 20% of their original weight (2 independent experiments with at least 10 animals per experimental group); *: *p* < 0.05 versus control group (no MSC injection).

Next, we compared the immunomodulatory potential of BM-MSC, UC-MSC, and MenSC, which were previously characterized according to the ISCT minimal criteria 27, by co-culturing them with PBMC isolated from healthy donors. Incubation with 2DG significantly impaired the intrinsic suppressive activity of human MSC (different donors) on CD4^+^ and CD8^+^ T cell proliferation (Figure 5A and Figure 5B, respectively). Conversely, incubation with oligomycin significantly increased their suppressive activity to comparable levels (all donors) (Figure 5A-B). Comparison of UC-MSC derived from different donors showed a great variability in their immunoregulatory activity towards CD4^+^ and CD8^+^ T cells (Figure 5C-D), as previously reported 28. Incubation with 2DG did not affect their immunoregulatory potential, conversely oligomycin significantly increased their suppressive capacity towards CD4^+^ and CD8^+^ T cells proliferation (Figure 5C-D). As pro-inflammatory cytokines might boost the immunosuppressive capacity of human MSC 22, we incubated UC-MSC with TNFα and IFNγ. However, we detected only a slight increase of the inhibition of CD4^+^ and CD8^+^ T cell proliferation, compared with UC-MSC_CTL_, unlike oligomycin that significantly increased this inhibitory effect (Figure S4A-B). Moreover, the percentage of IFNγ-producing CD4^+^ and CD8^+^ T cells was significantly reduced only when co-cultured with UC-MSC_oligomycin_ (Figure S4D-E), but not with UC-MSC_2DG_. We then studied the effect of metabolic reprogramming on the production of immune mediators (PD-L1, PGE_2_ and IDO) by human UC-MSC. In basal conditions, production of PD-L1 and PGE_2_ was comparable in UC-MSC_CTL_, UC-MSC_oligomycin_ and UC-MSC_2DG_. However, after stimulation with TNFα and IFNγ, production of PD-L1 (Figure 5E) and PGE_2_ (Figure 5F), but not IDO (Figure S5C), was significantly increased in UC-MSC_oligomycin_.

We also tested inhibitors of the electron transport chain, including inhibitors of complex III and IV, such as atovaquone and potassium cyanide (Figure S5F-G), and inhibitors of the ATP synthase complex, such as venturicidin, resveratrol and piceatannol. UC-MSC immunosuppressive activity was enhanced only by inhibitors of the ATP synthase complex (Figure S5H-I), suggesting a rather specific candidate target for therapeutic enhancement of MSC.

To determine whether oligomycin could improve UC-MSC therapeutic efficacy *in vivo*, we used a mouse model of GVHD to study the interaction between effector cells and UC-MSC, both of human origin. To this aim, we co-injected PBMC and UC-MSC_CTL_, UC-MSC_2DG_ or UC-MSC_oligomycin_, and then monitored weight loss in mice (Figure 5G). Survival rate was comparable in untreated mice and in mice that received UC-MSC_CTL_ or UC-MSC_2DG_. Conversely, survival rate was slightly improved in mice treated with UC-MSC_oligomycin_ (Figure 5H). Of note, we were not able to detect human proinflammatory cytokines such as TNFα or IFN-γ in the serum of GVHD mice.

### AMPK governs the immunosuppressive activity of glycolytic MSC

**Figure 6:**
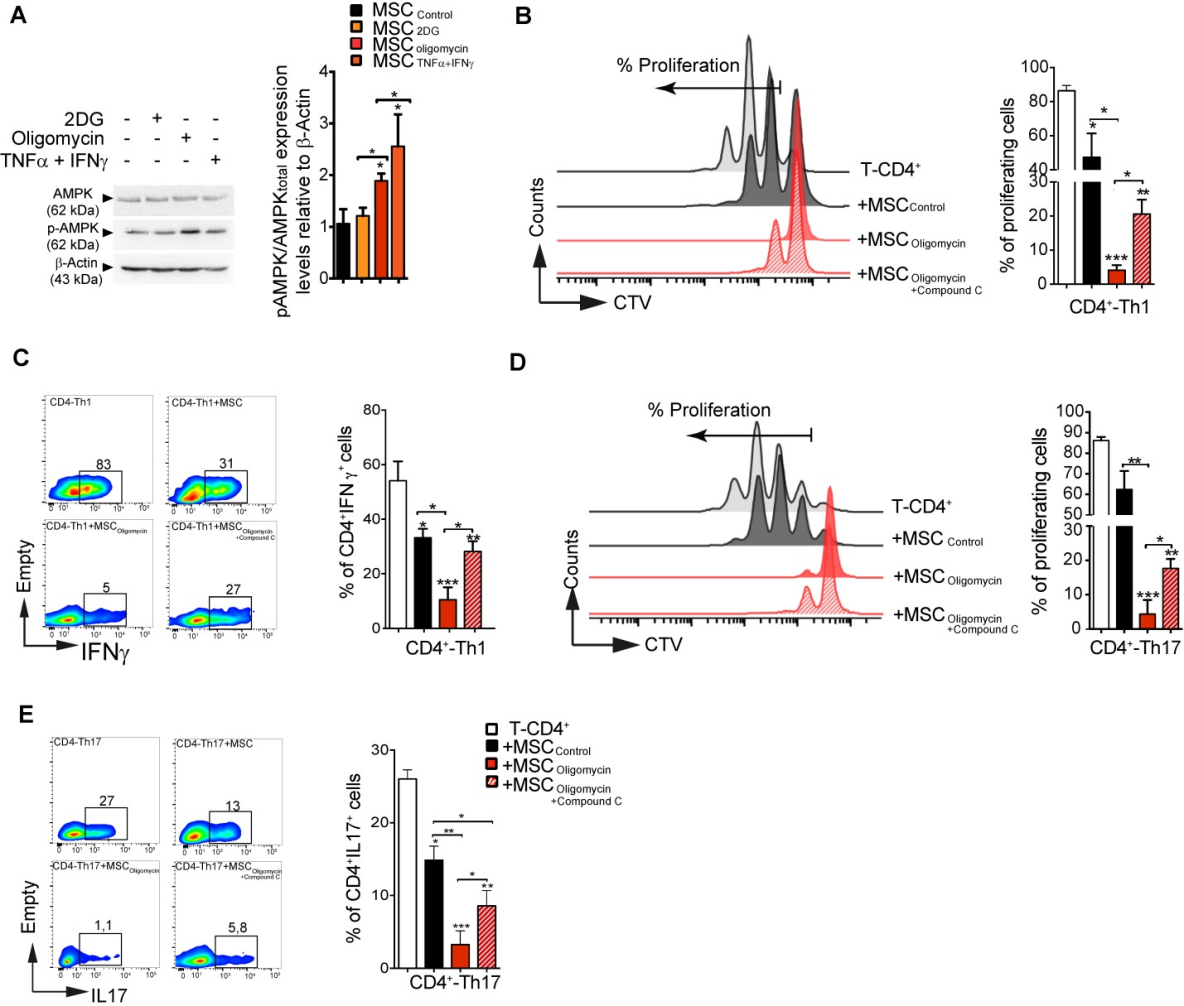
** AMPK regulates the suppressive activity of glycolytic MSCs.** The activity of AMPK (one of the main energy sensor in the cell) was evaluated after induction of the metabolic switch. Phosphorylated (p) AMPK and total AMPK **(A)** were quantified by western blotting. To determine AMPK role in glycolytic MSC suppressive potential towards pro-inflammatory Th1 and Th17 cells *in vitro*, naïve CD4^+^ T cells from Bl6 mice were labeled with CTV and induced to differentiate into Th1 **(B-C)** or Th17 **(D-E)** cells and cultured alone (white bar) or in the presence of murine MSC pre-incubated or not (black bar; Control) with oligomycin, with (stripy red bar) or without (red bar) compound C to inhibit AMPK activity. The T-cell proliferation and pro-inflammatory phenotype (IFNγ and IL17 production for Th1 and Th17, respectively) were evaluated by FACS. Results represent the mean ± SD of 4 independent experiments; *: *p* < 0.05, **: *p* < 0.01, ***: *p* < 0.001 (unpaired Kruskal-Wallis test). Unless otherwise indicated, comparisons were with CD4-Th1 or CD4-Th17 cultured alone.

AMPK has a very active role as cellular energy sensor and master controller of the adaptive response to changes in the metabolic requirements 29. Therefore, we asked whether AMPK signaling could be implicated in MSC metabolic switch induced by pro-inflammatory cytokines or by pharmacological treatment, and in the subsequent modulation of their suppressive activity. To this aim, we incubated murine MSC with TNFα and IFNγ, 2DG or oligomycin for 4 h before assessment of AMPK activation (i.e. AMPK phosphorylation on Thr172). AMPK phosphorylation level was significantly increased in MSC incubated with TNFα/IFNγ or oligomycin, but not with 2-DG (Figure 6A). Moreover, HIF1α expression was significantly increased in MSC_oligomycin_, and decreased in MSC_2DG_ (Figure S6), compared with MSC_CTL_. Therefore, to determine whether AMPK activity is implicated in the enhancement of the immunosuppressive function of MSC_oligomycin_, we co-cultured CD4^+^ T cells induced to differentiate into Th1 and Th17 cells with murine MSC_CTL_ or MSC incubated with oligomycin alone or in combination with compound C, a specific AMPK inhibitor. AMPK activity inhibition limited the enhancement of MSC suppressive activity (proliferation and phenotype) mediated by oligomycin towards both Th1 (Figure 6B-C) and Th17 cells (Figure 6D-E). These findings demonstrate that incubation with oligomycin or pro-inflammatory cytokines increases MSC immunosuppressive properties through AMPK signaling pathway activation.

## Discussion

This study provides solid evidence that MSC metabolic reprogramming significantly influences their therapeutic potential, and identified new molecular targets that control and connect both pathways. Specifically, we found that 1) priming MSC immunoregulatory and therapeutic potential using pro-inflammatory cytokines is associated with a glycolytic metabolic switch, 2) the pharmacologically-induced glycolytic switch in murine and human MSC substantially increases their anti-inflammatory and therapeutic effects, suggesting the ATP synthase complex is a specific target to achieve this goal, and 3) AMPK contributes to MSC glycolytic activity that drives their immunosuppressive activity.

MSC immunoregulatory effects are triggered by inflammation that is increased in response to tissue injury or during inflammatory or autoimmune diseases. *In vitro*, in response to pro-inflammatory factors, such IFNγ and TNFα, MSC release several factors that display immunoregulatory functions 22, 30-32, making them a candidate of choice for the treatment of disorders with an inflammatory phase. However, depending on the treatment and the disease phase, injected MSC can be exposed to different inflammatory stimuli that can modulate their immunosuppressive functions (increase or even loss) 33. These clinical parameters, in addition to the short period of time when injected MSC are detectable *in vivo*, narrow the therapeutic window of MSC 33. Here, we found that MSC activation by IFNγ and TNFα promotes MSC metabolic switch towards glycolysis. This is in line with recent studies showing that inflammation associated with pro-inflammatory cytokines induces a metabolic switch of human MSC towards a glycolysis-dependent metabolism 4, 6. Moreover, MSC co-priming with IFNγ and hypoxia enhances two times more their immunosuppressive properties via a glycolytic switch 34. This metabolic switch, leading to lactate production and inhibition of T cell proliferation, might enhance, extend and guarantee MSC therapeutic effects, regardless of the timing of injection.

During cancer progression, the metabolic competition between immune cells and tumor cells leads to a forced metabolic restriction of immune cells by cancer cells 35. Aerobic glycolysis is pivotal for the effector functions of T cells, and glucose consumption by tumors in a PD-L1-dependent manner substantially alters T cell function, promoting cancer progressions 35. Here, we found that TNFα/IFNγ-induced MSC glycolytic switch with activation of their immunosuppressive functions was associated with increased glucose uptake and lactate efflux. Moreover, activation of MSC_oligomycin_ by TNFα and IFNγ further enhanced PD-L1 expression level and MSC production of other immunosuppression mediators, such as PGE_2_.

To better characterize the highly immunosuppressive glycolytic MSC, we analyzed the expression profile of glucose transporters and metabolic enzymes upon incubation with pro-inflammatory cytokines. The mechanism and role of glucose uptake in MSC immunoregulatory properties have not been investigated yet. We showed that the significant increase of glucose uptake by MSC incubated with pro-inflammatory cytokines was associated with an increased expression of the glucose transporters GLUT1 and GLUT2. The GLUT transporter family includes fourteen members that are facilitative glucose transporters to allow glucose uptake 36. Glucose uptake by glucose transporters has a direct impact on cell functions and on the activity of metabolic enzymes. This was confirmed by the increased expression of PDHK1 and the active phosphorylated form of crucial enzymes of the glycolytic pathways, such as PFKB2 and LDH-A. Altogether, these data revealed the upregulation of most metabolic enzymes in MSC after the glycolytic switch, which is not surprising in glycolytic cells that exhibit immunoregulatory functions.

To further confirm the enhanced immunoregulatory activity of MSC_oligomycin_, we used two inflammatory experimental models: DTH for murine MSC and GVHD for human MSC because it allows studying the interactions between T lymphocytes and MSC 37, 38. MSC glycolytic reprogramming enhanced their immunosuppressive properties also *in vivo*. Indeed, MSC_oligomycin_ displayed enhanced capacity to inhibit T cell-mediated inflammation in the DTH model. This effect was associated with inhibition of Th17 and Th1 cells. Similarly, the survival rate in GVHD mice was slightly increased by treatment with MSC_oligomycin_, in line with studies showing that MSC are effective in the treatment of GVHD in preclinical and clinical trials 39-43. Since the GVHD experiment was finished when all the mice started to gain weight, we might be able to inadvertently eliminate data that could give us a better understanding of the therapeutic efficacy of MSC_oligomycin_. For this reason, it would be interesting to evaluate the effect of UC-MSC_oligomycin_ for longer periods of time on the GVHD murine model.

Finally, we identified a novel mechanism underlying MSC activation by pro-inflammatory cytokines. Indeed, by focusing on glycolytic metabolic pathways, we observed a significant increase of AMPK phosphorylation at Thr172 in MSC incubated with TNFα and IFNγ- and to a lower extent, with oligomycin. It has been reported that AMPK activity increases by more than 100-fold when AMPK is phosphorylated at Thr172 and by more than 1000-fold when phosphorylation at Thr172 is combined with allosteric regulation mediated by an increase in the AMP/ATP and ADP/ATP ratios 44. Moreover, AMPK promotes glucose uptake by phosphorylating thioredoxin-interacting protein (TXNIP), which controls GLUT1 translocation and cell-surface levels 45. Accordingly, our results showed an increased GLUT1 translocation to the membrane in MSC after TNFα and IFNγ stimulation. Moreover, we recently reported that TNFα and IFNγ stimulation significantly increases HIF-1α expression and translocation to the nucleus 46. Here, we observed decreased oxygen consumption and increased LDH-A phosphorylation that was associated with increased lactate production and glucose consumption. It has been reported that the LDH-A promoter contains HIF-1α binding sites 47, 48 and this might explain the increased expression of LDH-A. In addition, TNFα and IFNγ also increase LDH-A phosphorylation at Tyr10 that in turn upregulates LDH-A activity. LDH-A phosphorylation at Tyr10 regulates the NADH/NAD^+^ redox homeostasis, promoting glycolysis 49. HIF1α also increases MCT4 expression through hypoxia response elements found in the MCT4 promoter 50, 51, which is consistent with its proposed role in exporting lactic acid produced by glycolysis 52 and with MSC immunosuppression 46. Our results show that the metabolic switch induced by oligomycin enhances MSC immunosuppressive activity via an AMPK-dependent mechanism. Many evidences indicate that AMPK activation suppresses inflammatory responses through the inhibition of NF-κB and JAK-STAT signaling 53, 54. This is mediated by the activation of different pathways, such as FOXO, SIRT1 and p53 53, 55. Moreover, AMPK activation reduces macrophages proliferation induced by oxidized low-density lipoprotein 56. In addition, several reports indicate that AMPK can promote autophagy through phosphorylation and activation of unc-51-like autophagy-activating kinase 1 (ULK1), a serine/threonine kinase that triggers autophagy initiation 57, 58. Autophagy regulates MSC immunosuppressive properties towards CD4^+^ T cells through TGF-β1 and CXCL8 secretion 59 60, suggesting that AMPK sustains metabolism, and also immune responses. We recently showed *in vitro* and *in vivo* that HIF1α inhibition in MSC reduces their inhibitory potential towards Th1 and Th17 cell generation and their capacity to produce Treg cells, through a metabolic switch from glycolysis to OXPHOS 46. Although AMPK and HIF1α show some antagonistic properties (AMPK activates catabolic mechanisms to generate energy, while HIF promotes anabolic processes), it has been proposed that there is a reciprocal regulation between AMPK and HIF1α that is cell/tissue-specific and context-dependent 29. For example, AMPK regulates HIF1α nuclear accumulation that is critical for the generation of the hypoxia response 61. In addition, AMPK can phosphorylate and activate SIRT1 62 that in parallel stabilizes HIF1α through binding and deacetylation during hypoxia 62-65.

Altogether these data not only provide new insights into the molecular and metabolic pathways affected by bioenergetic reprogramming, but also confirm that MSC metabolic switch towards glycolysis enhances their immunoregulatory and therapeutic potential. Particularly, we found that ATP synthase inhibition is a specific strategy for functional enhancement of MSC. This strategy is reproducible, cheap and generates a very stable phenotype, compared with other approaches, such as exposure to proinflammatory cytokines or culture in hypoxic conditions. Thus, this study opens novel avenues for MSC-based immune-mediated disease therapy.

## Summary

The AMPK signaling pathway controls the metabolic status, immunoregulatory properties and therapeutic potential of mesenchymal stem cells.

## Supplementary Material

Supplementary figures and tables.
