# Supplementary material for: The ATP synthase inhibition induces an AMPK-dependent glycolytic switch of mesenchymal stem cells that enhances their immunotherapeutic potential
Source: Theranostics. 2021 Jan 1;11(1):445–60. doi: 10.7150/thno.51631 (PMC7681096; doi:10.7150/thno.51631)
Supplement: Supplementary file 1 — Supplementary figures and tables. [file thnov11p0445s1.pdf]

SUPPLEMENTARY MATERIAL

Figure S1

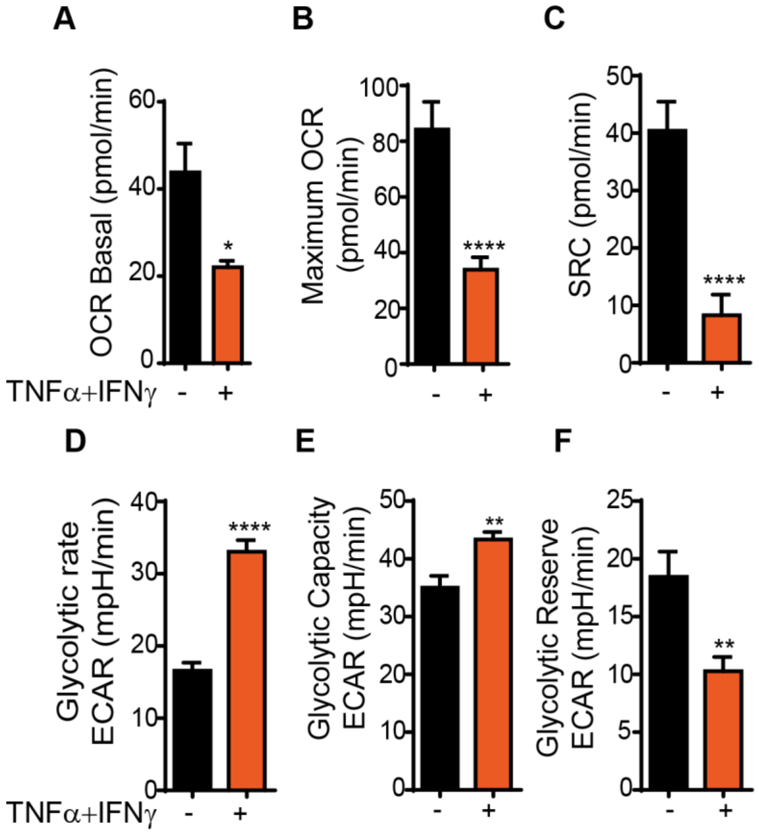

Figure S1. Pro-inflammatory cytokines induce a glycolytic metabolism in murine MSC. The metabolic status of murine MSC incubated or not with TNFα and IFNγ for 24 h was determined by analyzing the basal OCR **(A)**, the maximum OCR **(B)** and the SRC **(C)** using the Agilent Seahorse XF technology**.** The glycolytic rate was evaluated by quantifying ECAR in the medium **(D)**, the glycolytic capacity, **(E)** and the glycolytic reserve **(F)** using the Agilent Seahorse XF technology. Data are the mean ± SD of 5 independent experiments; *: $p < 0.05$, **: $p < 0.01$(unpaired Kruskal-Wallis test).

Figure S2

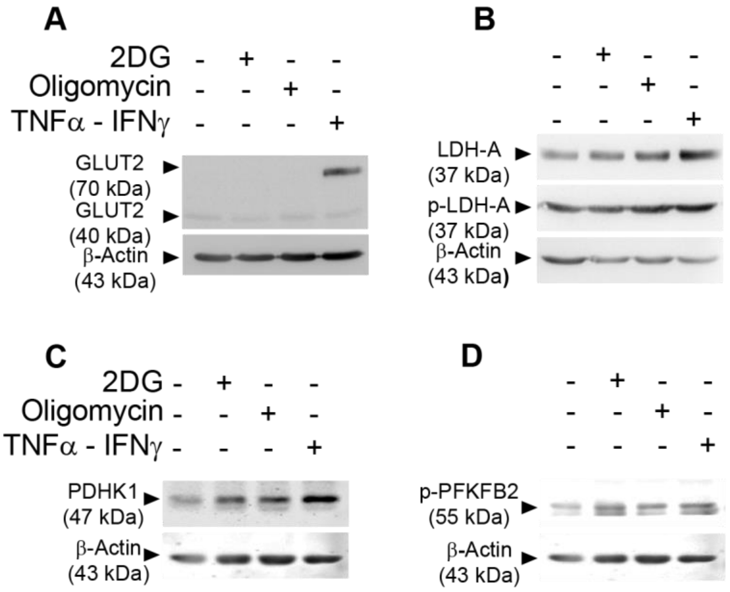

Figure S2. Representative western blot results for GLUT2 **(A),** LDH-A and phosphorylated LDH-A **(B),** PDHK1 **(C)** and phosphorylated PFKFB2 **(D)** in murine MSC incubated or not with TNFα and IFNγ, 2DG, or oligomycin, as indicated.

Figure S3

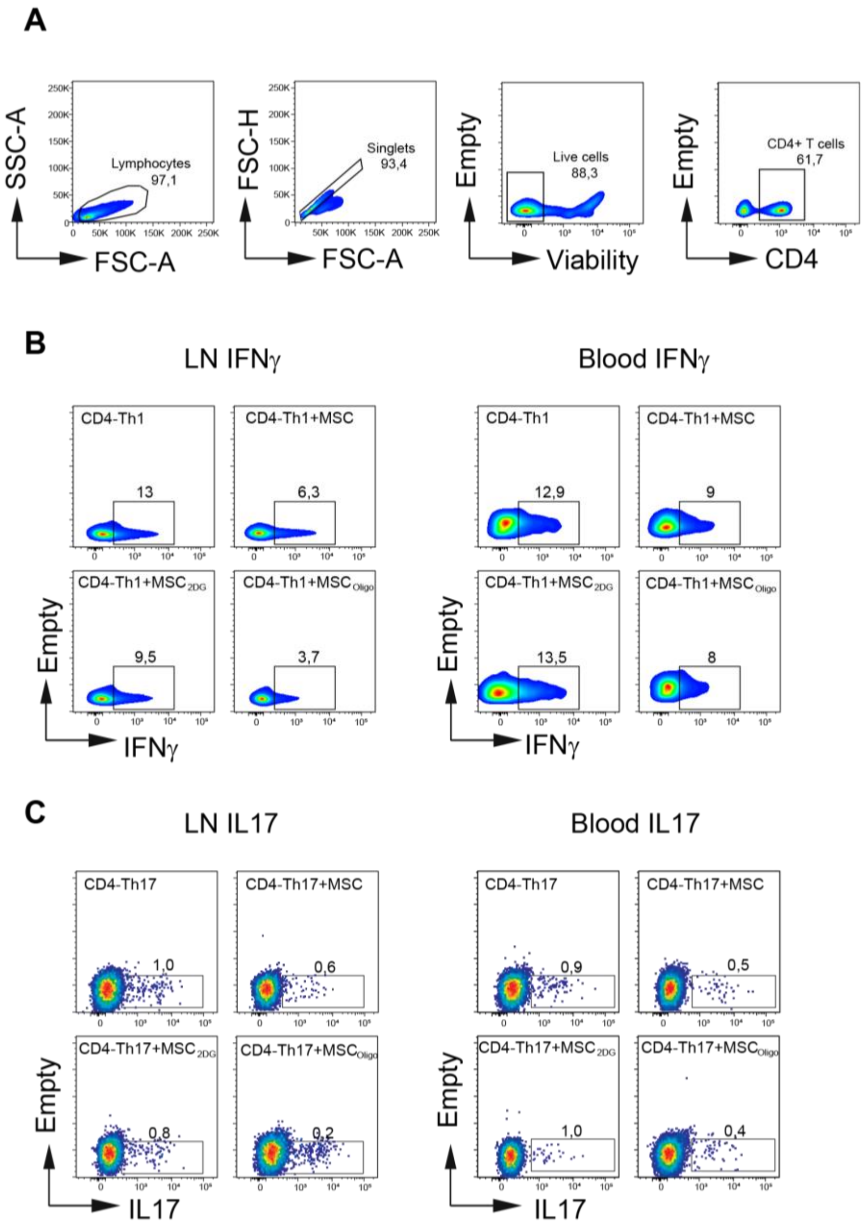

Figure S3. Representative dot plot and gate strategy for the DTH mouse model. At day 6 post-ovalbumin sensitization, DTH mice were euthanized, and peripheral blood and draining lymph nodes were harvested and CD4^{+} live single cells were analyzed by flow cytometry **(A)**. The generation of pro-inflammatory Th1 (CD4^{+}IFNγ^{+}) **(B)** and Th17 cells (CD4^{+}IL17^{+}) **(C)** was assessed in peripheral blood and draining lymph nodes of untreated DTH mice, or treated with control MSC, or MSCs incubated with 2DG or oligomycin.

Figure S4

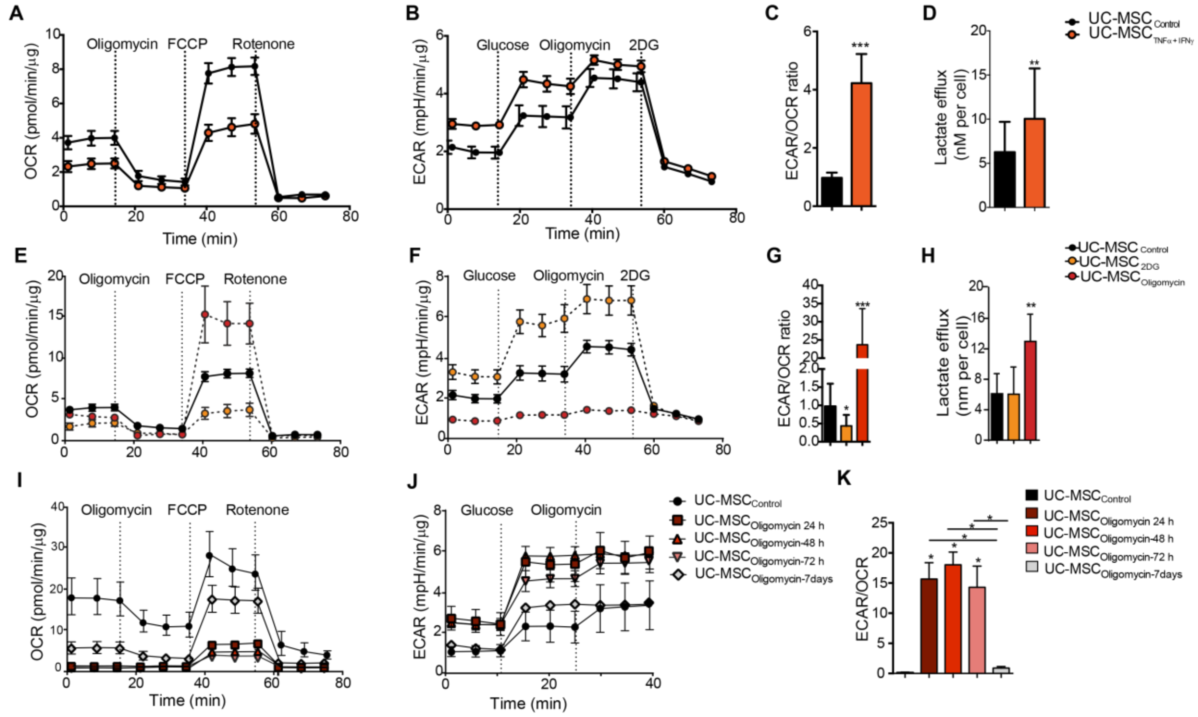

Figure S4. UC-MSC metabolic profile can be modified as observed in murine MSC. The metabolic profile of UC-MSC pre-incubated (orange) or not (black) with pro-inflammatory cytokines for 24 h was evaluated by measuring the **(A)** OCR, **(B)** ECAR and ECAR/OCR ratio **(C)** using the Agilent Seahorse XF technology. Lactate efflux was quantified in the supernatants of UC-MSC cultures stimulated or not with TNFα and IFNγ using the YSI biochemical analyzer **(D)**. Data are the mean ± SD of at least 3 independent experiments using 4 different UC-MSCs donors; **: $p < 0.01$, ***: $p < 0.001$ (unpaired Mann-Whitney test). The metabolic profile of UC-MSC pre-incubated or not (black) with 2DG (dark red) or oligomycin (red) for 24 h was evaluated by measuring the OCR **(E)**, ECAR **(F)** and the ECAR/OCR ratio **(G)** using the Agilent Seahorse XF technology**.** Lactate efflux **(H)** was measured in the supernatants with the YSI analyzer. The bioenergetics status of UC-MSC pre-incubated or not with oligomycin for 24 h was assessed at 1, 2, 3 and 7 days post-treatment by measuring the **(I)** OCR, **(j)** ECAR and ECAR/OCR ratio **(K)** using the

Agilent Seahorse XF technology. Data are the mean ± SD of at least 4 independent experiments; *: $p < 0.05$, **: $p < 0.01$, ***: $p < 0.001$, ****: $p < 0.0001$ (unpaired Kruskal-Wallis test).

Figure S5

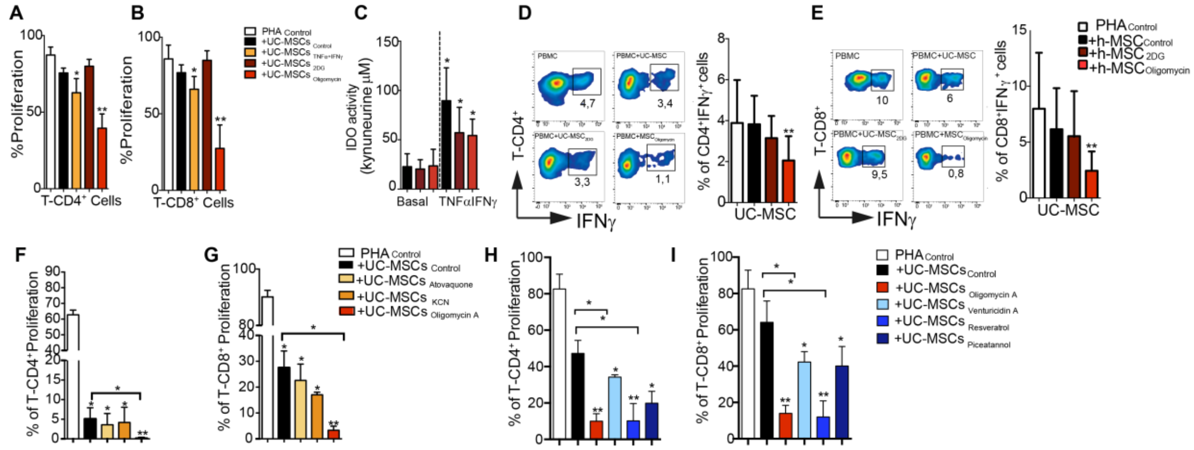

Figure S5. The enhanced functionality observed in glycolytic MSC is stronger when the F0F1-ATPase activity is inhibited. Human UC-MSC were incubated with oligomycin, 2DG or TNFα and IFNγ for 24 h and then cultured with PBMC for 4 days. The percentage of proliferating CD4$^+$ **(A)** and CD8$^+$ T cells **(B)** was quantified by FACS. **(C)** IDO activity was measured in the supernatants of UC-MSC pre-incubated or not with 2DG or oligomycin in the presence or absence of TNFα and IFNγ. IFNγ production by CD4$^+$ **(D)** or CD8$^+$ **(E)** T cells was quantified by FACS in PBMC co-cultured or not with UC-MSCs pre-incubated or not with 2DG or oligomycin. (**F**) Human UC-MSC were incubated or not with two electron transport chain inhibitors (atovaquone, and potassium cyanide) or oligomycin for 24 h and then cultured with PBMC for 4 days. The percentage of proliferating CD4$^+$ (F) and CD8$^+$ (G) T cells was determined by FACS. Human UC-MSCs were pre-incubated or not with different F0F1-ATPase inhibitors (resveratrol, piceatannol, venturicidin or oligomycin A) for 24 h and the percentage of proliferating CD4$^+$ **(I)** and CD8$^+$ **(J)** T cells was quantified by FACS. Data are the mean ± SD of at least 6 independent experiments; *: $p < 0.05$, **: $p < 0.01$ (unpaired Kruskal-Wallis test).

Figure S6

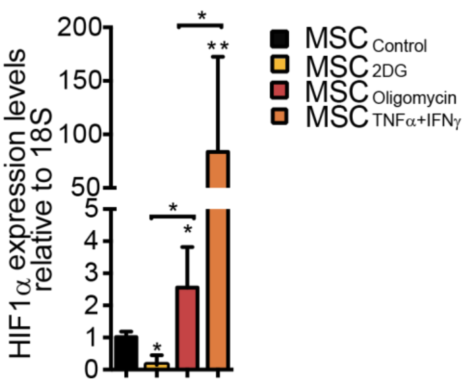

Figure S6. HIF1α expression level was quantified by qPCR in murine MSC pre-incubated or not with oligomycin, 2DG or TNFα and IFNγ. Data are the mean ± SD of at least 4 independent experiments; *: $p < 0.05$, **: $p < 0.01$ (unpaired Kruskal-Wallis test).

Table S1. List of the antibodies used for western blot studies.

Primary antibody	Dilution	Source	Supplier	Reference
GLUT2	1:1000	Rabbit	Alomone Labs	AGT022
MCT1	1:100	Rabbit	Merck Millipore	AB3538P
MCT4	1:100	Rabbit	Merck Millipore	AB3314P
β-actin	1:10000	Goat	Santa Crus Biotechnology	SC47778
β-actin (8H10D10)	1:20000	Mouse	Cell Signaling	3700
PDHK1 (C47H1)	1:1500	Rabbit	Cell Signaling	3820
Phospho-PFKFB2(Ser583)	1:1500	Rabbit	Cell Signaling	13064
AMPKα (F6)	1:1000	Mouse	Cell Signaling	2793
Phospho-AMPK (Thr172)	1:1000	Rabbit	Cell Signaling	2531
LDH-A	1:1500	Rabbit	Cell Signaling	2012
Phospho-LDH-A (Tyr10)	1:1500	Rabbit	Cell Signaling	8176
**Secondary antibody**	**Dilution**	**Source**	**Supplier**	**Reference**
AlexaFluor 680	1:40000	Rabbit	Invitrogen	A2110
AlexaFluor 750	1:40000	Mouse	Life Technologies	A21037
HRP Goat Anti-Mouse	1:5000	Mouse	Jackson Immunoresearch	115035146
HRP Goat Anti-Rabbit	1:5000	Rabbit	Jackson Immunoresearch	111035144

Table S2. List of the primers used for RT-qPCR

Gene	Forward	Reverse
18s	5’-GCCCGAAGCGTTTACTTTGA-3’	5’-TTGCGCCGGTCCAAGAATTT-3’
MCT1	5’-ACCAAGGCAGGGAAAGATAAG-3’	5’-AAAGCCTCTGTGGGTGAATAG-3’
MCT4	5’-CGCGGCTTTGTGCTTTAC-3’	5’-TGAAGAGGTAGACGGAGTAGG-3’
GPI	5’-CCATCAAGGTGGACGGCAAAGA-3’	5’-CCGTGATGGATTTGCCAGTGTAC-3’
HIF1α	5’-TGTGACCATGAGGAAATGAGAG-3’	5’-GGTTCACAAATCAGCACCAAG-3’
